# Identification of an integrated kinase-related prognostic gene signature associated with tumor immune microenvironment in human uterine corpus endometrial carcinoma

**DOI:** 10.3389/fonc.2022.944000

**Published:** 2022-09-07

**Authors:** Sitian Wei, Jun Zhang, Rui Shi, Zhicheng Yu, Xingwei Chen, Hongbo Wang

**Affiliations:** ^1^ Department of Gynecology and Obstetrics, Union Hospital, Tongji Medical College, Huazhong University of Science and Technology, Wuhan, China; ^2^ Department of Industrial engineering, Tsinghua University, Beijing, China

**Keywords:** kinase, prognosis, gene signature, tumor immune microenvironment, uterine corpus endometrial carcinoma

## Abstract

In the worldwide, uterine corpus endometrial carcinoma (UCEC) is the sixth most common malignancy in women, and the number of women diagnosed is increasing. Kinase plays an important role in the occurrence and development of malignant tumors. However, the research about kinase in endometrial cancer is still unclear. Here, we first downloaded the gene expression data of 552 UCEC patients and 23 healthy endometrial tissues from The Cancer Genome Atlas (TCGA), obtained 538 kinase-related genes from the previous literature, and calculated 67 differentially expressed kinases. Gene Ontology (GO) and Kyoto Encyclopedia of Genes and Genomes (KEGG) were referenced to identify multiple important biological functions and signaling pathways related to 67 differentially expressed kinases. Using univariate Cox regression and Least absolute shrinkage and selection operator (LASSO), seven kinases (ALPK2, CAMKV, TTK, PTK6, MAST1, CIT, and FAM198B) were identified to establish a prognostic model of endometrial cancer. Then, patients were divided into high- and low-risk groups based on risk scores. Receiver operating characteristic (ROC) curves were plotted to evaluate that the model had a favorable predictive ability. Kaplan–Meier survival analysis suggested that high-risk groups experienced worse overall survival than low-risk groups. qRT-PCR and ISH assays confirmed the consistency between predicted candidate genes and real sample contents. CIBERSORT algorithm and ssGSEA were adopted to investigate the relationship between this signature and tumor immune microenvironment, and revealed that in low- and high-risk groups, the types of tumor-infiltrating immune cells and the immune cell-related functions were significantly different. In summary, a seven-gene signature risk model has been constructed, and could accurately predict the prognosis of UCEC, which may offer ideas and breakthrough points to the kinase-associated development of UCEC.

## Introduction

Uterine corpus endometrial carcinoma (UCEC) is one of the common malignant tumors of the female reproductive system, which is a kind of malignant epithelial tumor occurring in the endometrium. According to the latest global cancer burden data released by the international agency for research on cancer (IARC) of the World Health Organization in 2020, UCEC is the sixth most common female cancer, with 417,000 new cases and 97,000 deaths in 2020 (1). In recent years, there is a growing number of UCEC patients, and the age tends to be younger. Most UCEC patients can be diagnosed early and can be treated by surgery, radiotherapy, chemotherapy, molecular targeted therapy, and other methods, with a good prognosis (2, 3). However, although there are more and more therapeutic schedules available (4, 5), the results of advanced, poorly differentiated, or special types of UCEC are still unsatisfactory (6, 7). Therefore, the identification of potential predictors is urgently required to improve the prognosis for patients with UCEC.

Kinases are enzymes that transfer phosphate groups from high-energy donor molecules (such as ATP) to specific target molecules (substrates). This process is called phosphorylation. The human kinome comprises 538 kinases, which are encoded by about 2% of the human genome and play an important role in catalyzing protein phosphorylation (8). Protein kinases are distributed throughout the nucleus, mitochondria, microsomes, and cytoplasm in cells, which covalently bind to the hydroxyl groups of some serine, threonine, or tyrosine residues in specific protein molecules through catalyzing phosphate groups, thereby changing the conformation and activity of proteins and enzymes. Protein kinases are generally divided into three categories. ① Substrate-specific protein kinases: such as phosphorylase kinase, pyruvate dehydrogenase kinase, etc. ② Cyclic nucleotide-dependent protein kinases: such as cAMP protein kinase and cGMP protein kinase. ③ Other protein kinases: such as histone kinases. These kinases play a wide range of roles in cell signal transduction and complex life activities, including immunity, cell growth and division, and metabolism. Other different kinases act on small molecules (lipids, sugars, amino acids, nucleosides, etc.) either to issue a call or prepare for various biochemical reactions in metabolism.

Protein kinases regulate the key processes of almost all cell activities. Therefore, dysfunctions, including overexpression, relocation, point mutations, or upstream signal transduction disorders (9), are likely to lead to the occurrence and development of a variety of diseases, such as tumors (10). In fact, the first proto-oncogene c-Src identified in 1978 has the ability to encode a non-receptor tyrosine kinase (11). For over several decades, the study of kinases as drug targets has great potential, and kinases have been the focus of pharmaceutical drug discovery efforts. By now, the US Food and Drug Administration (FDA) has approved 48 small-molecule kinase inhibitors. Exploring the functions of kinases not only helps to promote the development of cancer biology but also the development of agonists and antagonists of these enzymes can provide the possibility of targeted treatment for diseases.

The relationship between kinome and the occurrence and development of cancer has been reported in early studies. More than 450 kinases are associated with the development or progression of diseases (12). Of these, 448 are linked to various genetic and signaling cancer hallmarks, while 230 potentially play a role in the development of other diseases and developmental disorders (13). Previous studies have mostly focused on the role of a single kinase in the development and treatment of endometrial cancer (14, 15). However, there is no large-scale data mining study to analyze the effect of kinome on cancer progression and prognosis. In the present study, we analyzed the transcriptome data of endometrial cancer samples downloaded from the public database of The Cancer Genome Atlas (TCGA) and constructed a prognostic multigene signature with even protein kinase-related genes, which can accurately evaluate the prognostic risk of UCEC patients. We further evaluated the relationships between the gene signature and tumor-infiltrating immune cells (TICs) infiltration to explore the potential value of the prognostic model.

## Methods

### Patient information and databases

Transcript information of endometrial cancer samples was downloaded from The Cancer Genome Atlas (TCGA, https://portal.gdc.cancer.gov/), with 23 normal samples and 552 endometrial cancer samples. The clinical information on these patients was downloaded from the UCSC Xena database (https://xenabrowser.net/heatp/). Then, 538 kinase-related genes were retrieved from the previous literature (16) and are provided in **Supplementary Table S1**.

### Screening and enrichment analysis of differentially expressed genes

By sorting and analyzing the transcriptome data of endometrial cancer samples in the TCGA database, the mRNA expression matrix of UCEC samples was obtained. The Limma software package in R statistical software was adopted to filtrate differentially expressed genes associated with kinome (FDR Filter = 0.05, logFC filter = 1). The ClusterProfiler, org. Hs.eg.db, and ggplot2 packages in R were applied to perform enrichment analysis of all the differentially expressed kinases using the Gene Ontology (GO) and Kyoto Encyclopedia of Genes and Genomes (KEGG) databases in order to discover the main biological characteristics of these genes (17).

### Training and validation of the prognostic model

Using the “caret” R software package, the TCGA-UCEC cohort was randomly divided into a training set and a verification set, and we obtained a training set with 271 samples and a verification set with 271 samples. The risk score of each sample in both sets was calculated and the samples were stratified into high-risk and low-risk groups based on the median value of the risk score.

### Construction of a prognostic kinase-related gene signature

Kinases differentially expressed in UCEC had performed a univariate Cox regression. Using the “survival” package to plot univariate Cox regression analyses; a P value less than 0.05 was considered a significant difference threshold. In order to simplify the parameters of the model and minimize the risk of overfitting, the Least Absolute Shrinkage and Selection Operator (LASSO) regression analysis was carried out for variable selection and shrinkage with the “glmnet” R package (18). Then, the genes acquired by LASSO regression were treated with multivariate Cox regression, and the multivariate regression coefficient of every gene was calculated, on which a risk scoring equation was constructed. Besides, survival curves were plotted with the “survminer” R package, and the time‐dependent receiver operating characteristic (ROC) curve evaluating the accuracy of the prognostic model was constructed using the “timeROC” R package (19).

### The mRNA expression analysis of kinase-related gene signature

In order to study the expression of the kinase-related gene signature at the mRNA level, the quantitative real-time polymerase chain reaction (qRT-PCR) was performed in tissue samples. A total of seven paired EC and adjacent normal tissues of patients who underwent surgery or biopsy in the Department of Gynecology, Union Hospital Affiliated to Tongji Medical College, Huazhong University of Science and Technology (Wuhan, China) from September 2019 to March 2021. All patients had complete clinical data and did not receive immunotherapy, chemotherapy, or radiotherapy. This study was approved by the Ethics Committee of Tongji Medical College, Huazhong University of Science and Technology (No. 2022-S017).

The tissues were harvested with the RNAiso reagent (Takara, Japan). The extraction of RNA was performed according to the manufacturer’s manual. The fold changes of RNA transcripts were calculated by the 2−ΔΔCt method and glyceraldehyde 3-phosphate dehydrogenase (GAPDH) was utilized as the endogenous control. The primer sequences used were shown in **Supplementary Table S2**.

### Seven kinase proteins expression verification

Immunohistochemical staining maps about protein levels of seven kinase-related gene signatures in normal endometrium and endometrial cancer were acquired from the Human Protein Atlas (HPA, https://www.proteinatlas.org/). The IHC staining scores were evaluated by two independent observers blinded to the corresponding patients based on the staining intensity (SI) and the percentage of immunoreactive cells (PR). The SI score was calculated from 0 to 3: 0 = no staining; 1 = weak staining; 2 = moderate staining; and 3 = strong staining. The PR was scored from 1 to 4: 1 = 0–25%; 2 = 26–50%; 3 = 51–75%; and 4 = 75–100%. The PR and the SI were multiplied to produce a weighted score for each patient. A score of 8–12 was defined as a high expression level, and a score of 0–7 was defined as low expression.

### Statistical analysis

All statistical analyses were performed using the R software (Version 3.6.1) and GraphPad Prism 8 software (GraphPad Software, San Diego, CA, USA). Measurement data were presented by (mean ± SEM), and every assay was performed in triplicate. The unpaired t-test was used to compare the ISH score and mRNA expression level between the normal endometrial tissue and UCEC tissue. P < 0.05 was considered statistically significant. ns: not significant, *P < 0.05, **P < 0.01, ***P < 0.001, ****P < 0.0001.

## Results

### Differentially expressed kinases

**Figure 1:**
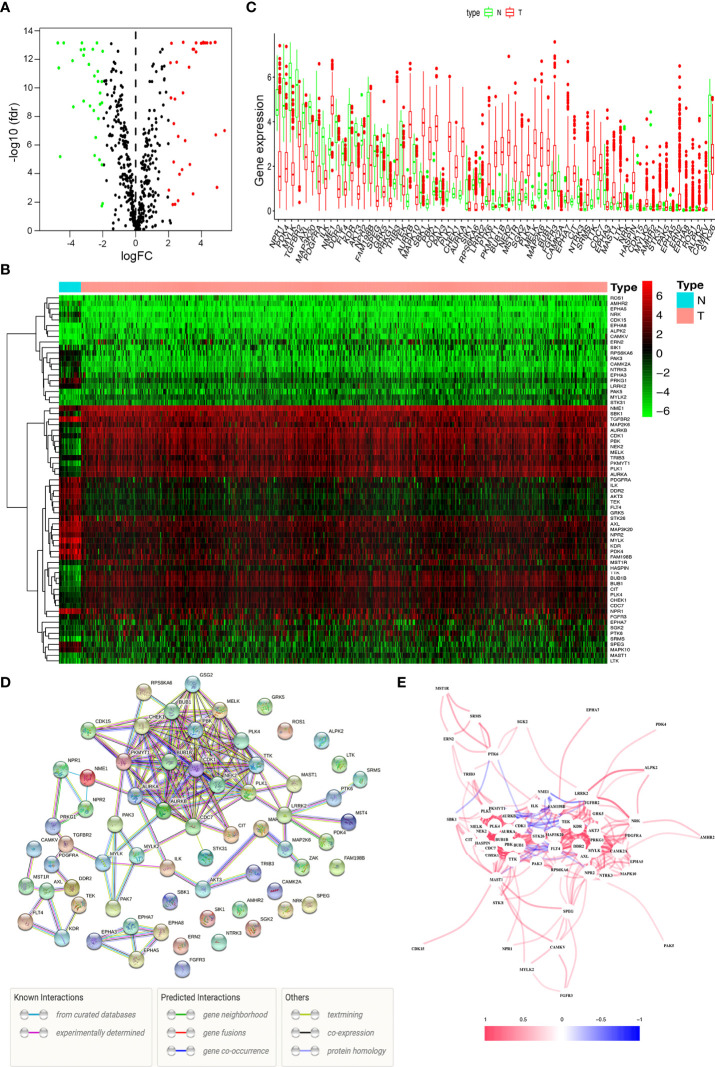
Differentially expressed kinases. **(A)** The volcano plot for the 475 kinase-related genes from the TCGA data portal. **(B)** Heat map of 36 up-regulated and 31 down-regulated kinases. **(C)** The expression patterns of 67 kinase-related genes in endometrial cancer and normal endometrial tissues. **(D)** Construction of the PPI network downloaded from the STRING database about the candidate genes. **(E)** The correlation network of 67 kinase-related genes.

We analyzed the expression of 538 kinase-related genes in 552 endometrial cancer tissues and 23 non-tumor tissues from TCGA database using Wilcoxon signed-rank test in R. According to the standard of |log2FC| > 1 and FDR < 0.05, we obtained 67 differentially expressed kinases, including 36 up-regulated genes and 31 down-regulated genes (**Supplementary Table S3**). The volcano map (**Figure 1A**), heat map (**Figure 1B**), and boxplot (**Figure 1C**) were drawn with differential genes. The color types and depth represent the level of gene expression. The protein-protein interaction (PPI) network revealed correlations between these genes, and 15 genes did not form part of the network (**Figure 1D**). The correlations between these candidate genes were complicated (**Figure 1E**), such as PTK6 had a similar positive correlation with SRMS, SGK2, MST1R, etc., and showed a negative coexpression correlation with SBK1.

### GO and KEGG enrichment analysis of differentially expressed kinases

**Figure 2:**
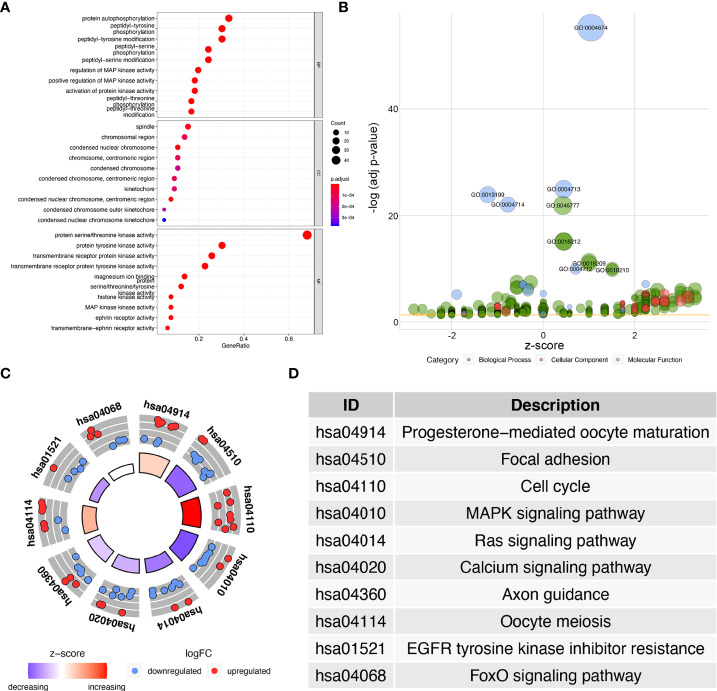
GO and KEGG enrichment analysis of differentially expressed kinases. **(A)** Dot plot of significant GO terms of 67 differentially expressed kinases. **(B)** Bubble plot of enriched GO terms. **(C)** Circle plot of KEGG enrichment analysis of 67 differentially expressed kinases. **(D)** The table lists the name of each KEGG term.

The 67 differentially expressed kinase-related genes were analyzed by GO function enrichment and KEGG pathway enrichment (**Supplementary Tables S4**, **5**), and the enrichment results were visualized to understand the biological functions of these genes. GO function enrichment analysis results showed these kinases were primarily involved with the protein autophosphorylation, peptidyl-tyrosine phosphorylation, peptidyl-tyrosine modification, spindle, chromosomal region, protein serine/threonine kinase activity, protein tyrosine kinase activity (**Figures 2A, B**). The results of the KEGG pathway enrichment analysis indicated that the differentially expressed kinases were related to progesterone-mediated oocyte maturation, focal adhesion, cell cycle, MAPK signaling pathway, Ras signaling pathway, calcium signaling pathway, axon guidance, oocyte meiosis, EGFR tyrosine kinase inhibitor resistance, and FoxO signaling pathway (**Figures 2C, D** and**Table 1**).

**Table 1:** KEGG enrichment results for differentially expressed kinases.

ID	Description	P	geneID
hsa04914	Progesterone-mediated oocyte maturation	6.95E-08	AKT3/MAPK10/CDK1/PLK1/AURKA/RPS6KA6/PKMYT1/BUB1
hsa04510	Focal adhesion	1.04E-07	MYLK/PDGFRA/ILK/FLT4/KDR/AKT3/MAPK10/PAK3/MYLK2/PAK5
hsa04110	Cell cycle	3.61E-07	CDK1/PLK1/CHEK1/PKMYT1/BUB1B/BUB1/TTK/CDC7
hsa04010	MAPK signaling pathway	3.97E-07	TGFBR2/MAP3K20/PDGFRA/FLT4/KDR/AKT3/TEK/MAPK10/RPS6KA6/MAP2K6/FGFR3
hsa04014	Ras signaling pathway	3.95E-06	PDGFRA/FLT4/KDR/AKT3/TEK/MAPK10/PAK3/FGFR3/PAK5
hsa04020	Calcium signaling pathway	5.22E-06	MYLK/PDGFRA/FLT4/KDR/MST1R/FGFR3/CAMK2A/NTRK3/MYLK2
hsa04360	Axon guidance	5.83E-06	ILK/PAK3/CAMK2A/EPHA7/EPHA3/PAK5/EPHA5/EPHA8
hsa04114	Oocyte meiosis	6.64E-06	CDK1/PLK1/AURKA/RPS6KA6/PKMYT1/BUB1/CAMK2A
hsa01521	EGFR tyrosine kinase inhibitor resistance	6.94E-05	AXL/PDGFRA/KDR/AKT3/FGFR3
hsa04068	FoxO signaling pathway	7.68E-05	TGFBR2/AKT3/MAPK10/PLK1/SGK2/PLK4

### Survival-related kinases and the prognostic model

**Figure 3:**
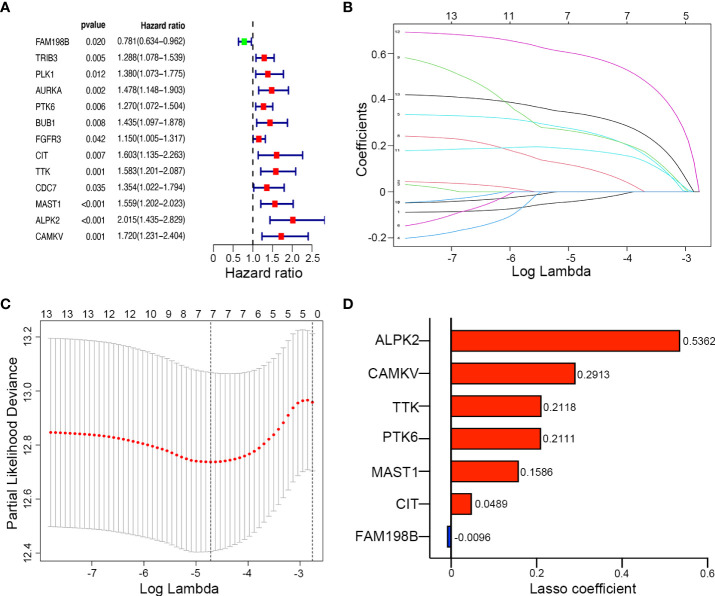
Construction of a kinase-related gene signature prognostic model in UCEC. **(A)** The forest map of univariate Cox regression analysis results of 9 prognostic kinase genes. **(B)** The LASSO coefficient profile plot of the 9 survival-related kinases. **(C)** LASSO regression analysis of kinase-associated survival genes based on the optimal tuning parameter (λ). **(D)** The distribution of LASSO coefficients of the gene signature.

We investigated the prognostic role of 67 differentially expressed kinase-related genes in 271 training UCEC patients. Based on univariate Cox regression analysis, 13 kinases associated with the prognosis of endometrial cancer were obtained (**Figure 3A**), including FAM198B, TRIB3, PLK1, AURKA, PTK6, BUB1, FGFR3, CIT, TTK, CDC7, MAST1, ALPK2, and CAMKV (P < 0.05). Only one prognosis-related kinase (FAM198B) was considered a protective factor (the minimum value of 95% CI was smaller than 1). In contrast, the high expression of the remaining twelve genes (TRIB3, PLK1, AURKA, PTK6, BUB1, FGFR3, CIT, TTK, CDC7, MAST1, ALPK2, and CAMKV) presented worse survival.

LASSO regression analysis was then conducted to establish a prognostic model using the expression profile of the 13 genes mentioned above. The complexity degree of LASSO regression is determined by the parameter lambda (λ). Based on the optimal value of λ, 7 candidate kinases were still identified (ALPK2, CAMKV, TTK, PTK6, MAST1, CIT, and FAM198B) by LASSO regression (**Figures 3B, C**). The distribution of LASSO coefficients of the gene signature were shown in **Figure 3D**.

Meanwhile, Kaplan–Meier survival analysis was performed for each gene separately, and survival curves were plotted (**Supplementary Figure S1**.). Consistent with the above, UCEC patients with high expression of risk genes (ALPK2, CAMKV, TTK, PTK6, MAST1, and CIT) have a poor prognosis, while patients with high expression of FAM198B have a better prognosis. The risk score of kinase-related genes for OS = (0.5362 × ALPK2) + (0.2913 × CAMKV) + (0.2118 × TTK) + (0.2111 × PTK6) + (0.1586 × MAST1) + (0.0489 × CIT) + (-0.0096 × FAM198B). Therefore, 7 kinase-related genes were chosen to build the predictive model consisting of Alpha Kinase 2 (ALPK2), CaM Kinase Like Vesicle Associated (CAMKV), TTK Protein Kinase (TTK), Protein Tyrosine Kinase 6 (PTK6), Microtubule Associated Serine/Threonine Kinase 1 (MAST1), Citron Rho-Interacting Serine/Threonine Kinase (CIT), and Family With Sequence Similarity 198 Member B (FAM198B).

### Validation of prognostic kinase-related risk signature in UCEC

**Figure 4:**
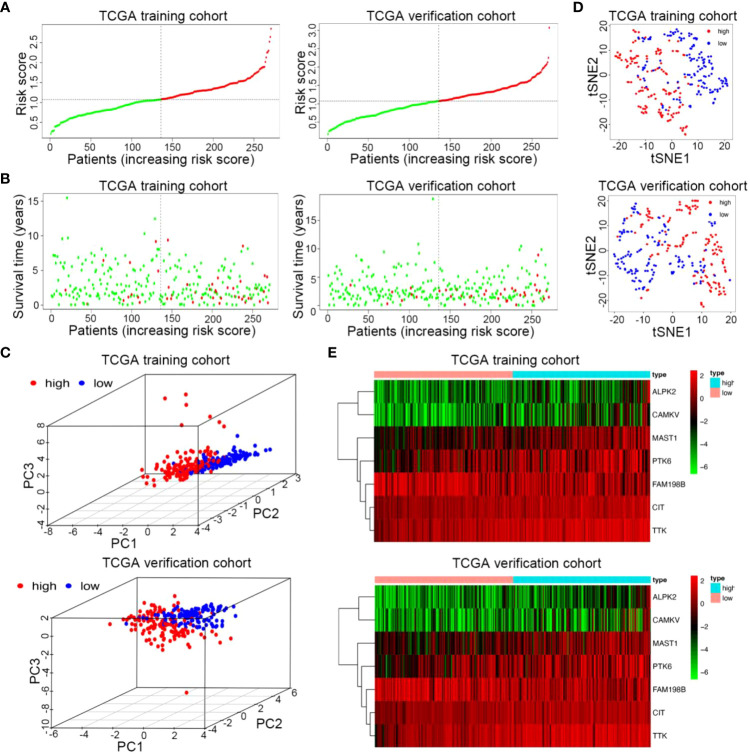
Risk curve and heatmap of risk genes in the training and verification sets. **(A)** Distribution of risk score of the prognostic kinase-related risk gene signature in the training and verification cohort. **(B)** PCA plot of the TCGA train and verification cohorts. **(C)** t-SNE analysis of the two TCGA cohorts. **(D)** Scatterplots of UCEC patients with different survival statuses in the training set and verification set. **(E)** The heatmap of 7 kinase-related genes in UCEC between high-risk and low-risk patients in the training and verification sets.

We used the median risk value to divide the training and verification cohorts into a high-risk group (n = 135) and a low-risk group (n = 136) (**Figure 4A**). PCA and t-SNE analysis indicated the patients in different risk subgroups were distributed in two discrete directions (**Figures 4B, C**). As shown in **Figure 4D**, in both sets, as the patient’s risk value increased, patient mortality increased significantly, and survival time dramatically shortened. The heatmap showed expression of 6 risk genes, including ALPK2, CAMKV, TTK, PTK6, MAST1, and CIT, were highly up-regulated in the high-risk groups, except for 1 protective gene (FAM198B) (**Figure 4E**). The results from the training and verification sets were internally consistent.

### Prognostic value of the kinase-related genes risk signature in UCEC

**Figure 5:**
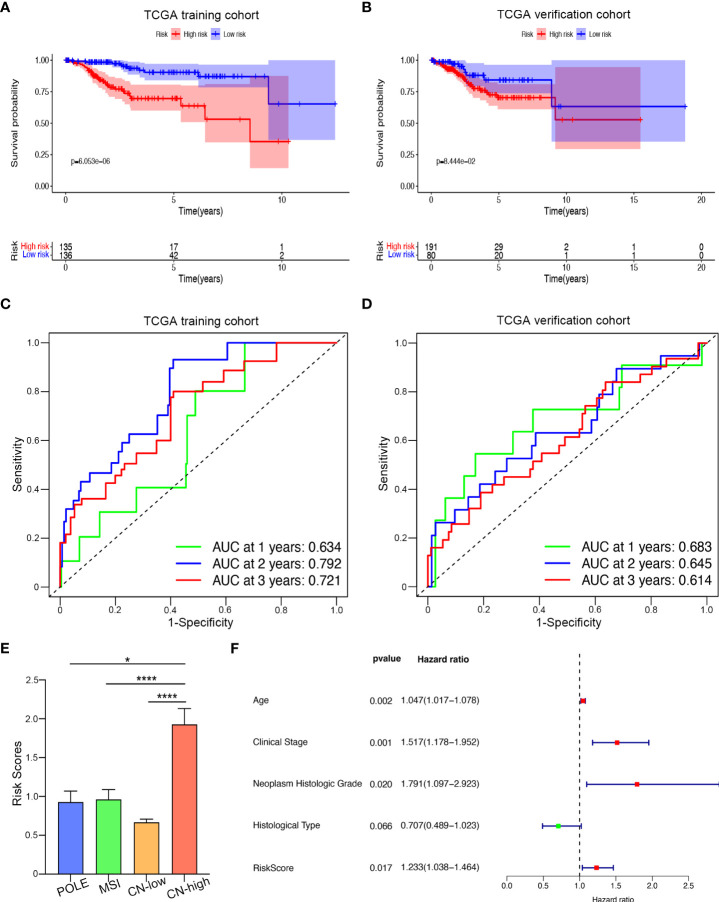
Prognostic value of the kinase-related risk signature in UCEC. **(A)** Kaplan-Meier curves of the high-risk and low-risk UCEC patients in the training set. **(B)** Kaplan-Meier plotter of OS for UCEC patients in the verification group. **(C)** Time-dependent ROC curves for predicting one-year, three-year, and five-year survival in the training cohort; **(D)** AUC of time-dependent ROC curves for predicting one-year, three-year, and five-year survival in the testing cohort; **(E)** Distributions of risk scores among four different subtypes of UCEC. **(F)** The forest plot visualized the HRs of clinicopathological criteria identified by multivariate Cox analysis.*P<0.005,****P<0.0001

Afterward, we drew Kaplan–Meier survival curves. As shown in **Figures 5A, B**, both in the training and validation cohorts, the overall survival (OS) of the high-risk group was shorter, and patients in the high-risk group were more likely to encounter death earlier (P < 0.05). To evaluate the predictive efficiency of the kinase-related risk signature in the 1-, 3-, and 5-year survival rates, the ROC curve was performed, where the area under the curve (AUC) was 0.702 for one-year survival, 0.709 for three-year survival, and 0.716 for five-year survival, respectively, indicating a high predictive value (**Figure 5C**). This was further validated in the verification set (**Figure 5D**).

Next, we examined the relationship between our prognostic signature and the molecular classification of UCEC. As **Figure 5E** showed, patients with copy-number-high (CN-high) had the highest risk score, compared with the risk score of patients with polymerase epsilon (POLE) ultramutated, microsatellite-instable (MSI), or copy-number-low (CN-low). We then analyzed the relationship between risk score and clinicopathological parameters in UCEC (**Table 2**). The results indicated that when UCEC patients with older age, higher FIGO stage, higher neoplasm Histologic Grade, or worse histological type, they might suffer higher risk score. However, BMI seemed not to affect the risk score. Furthermore, we performed the COX analysis again and included risk score, age, FIGO stage, histologic grade, and histological type (**Figure 5F**). Like traditional endometrial carcinoma prognostic markers, the prognostic score acquired by our kinase-related prognostic model was also an independent and feasible prognostic factor for UCEC, with an HR of 1.2337 (p = 0.017).

**Table 2:** Clinical and pathological characteristics of high- and low-risk patients.

Parameter	Risk			χ^2^	p value
	Number	Low (n = 183)	High (n = 184)		
Age (years)					
<60	131	81	50	11.672	< 0.001
≥60	236	102	134
Clinical Stage					
FIGOI	251	146	105	23.593	< 0.001
FIGOII	25	8	17
FIGOIII	74	26	48
FIGOIV	17	3	14
Neoplasm Histologic Grade					
G 1	88	68	20	74.957	< 0.001
G 2	103	68	35
G 3	176	47	129
Histological Type					
Endometrioid endometrial adenocarcinoma	301	177	124	54.778	<0.001
Mixed serous and endometrioid	13	3	10
Serous endometrial adenocarcinoma	53	3	50
BMI					
<30	142	64	78	2.129	0.164
≥30	225	119	106

### Validation of prognostic kinase-related genes at the mRNA and protein level

**Figure 6:**
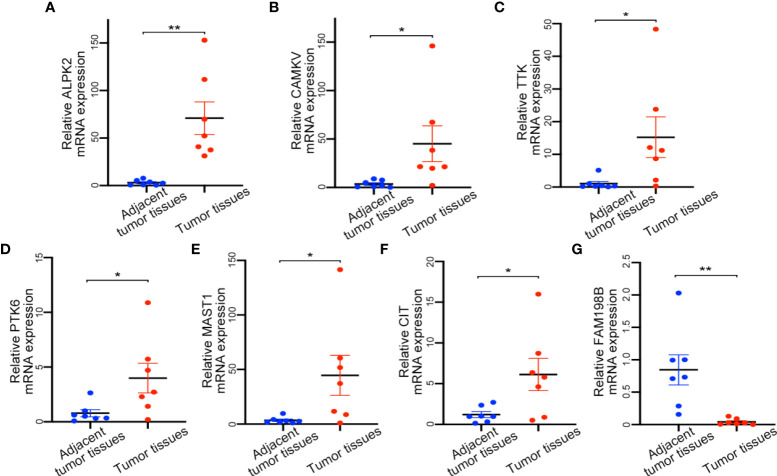
Expression of detection of kinase-related gene signature by qRT-PCR. **(A)** ALPK2 mRNA expression level. **(B)** CAMKV mRNA expression level. **(C)** TTK mRNA expression level. **(D)** PTK6 mRNA expression level. **(E)** MAST1 mRNA expression level. **(F)** CIT mRNA expression level. **(G)** FAM198B mRNA expression level. *P<0.05,**P<0.01.

After that, we used tissue samples to confirm whether screened 7 kinase-related genes were consistent with bioinformatic analysis. The tumor tissues and adjacent normal tissues were from 7 patients with UCEC, and the mRNA expression of these genes was validated by qRT-PCR. The results revealed that all candidate genes were significantly different between normal and UCEC tissues (**Figure 6**). Among them, ALPK2, CAMKV, TTK, PTK6, MAST1, and CIT were upregulated, and FAM198B was remarkably downregulated in tissue samples.

**Figure 7:**
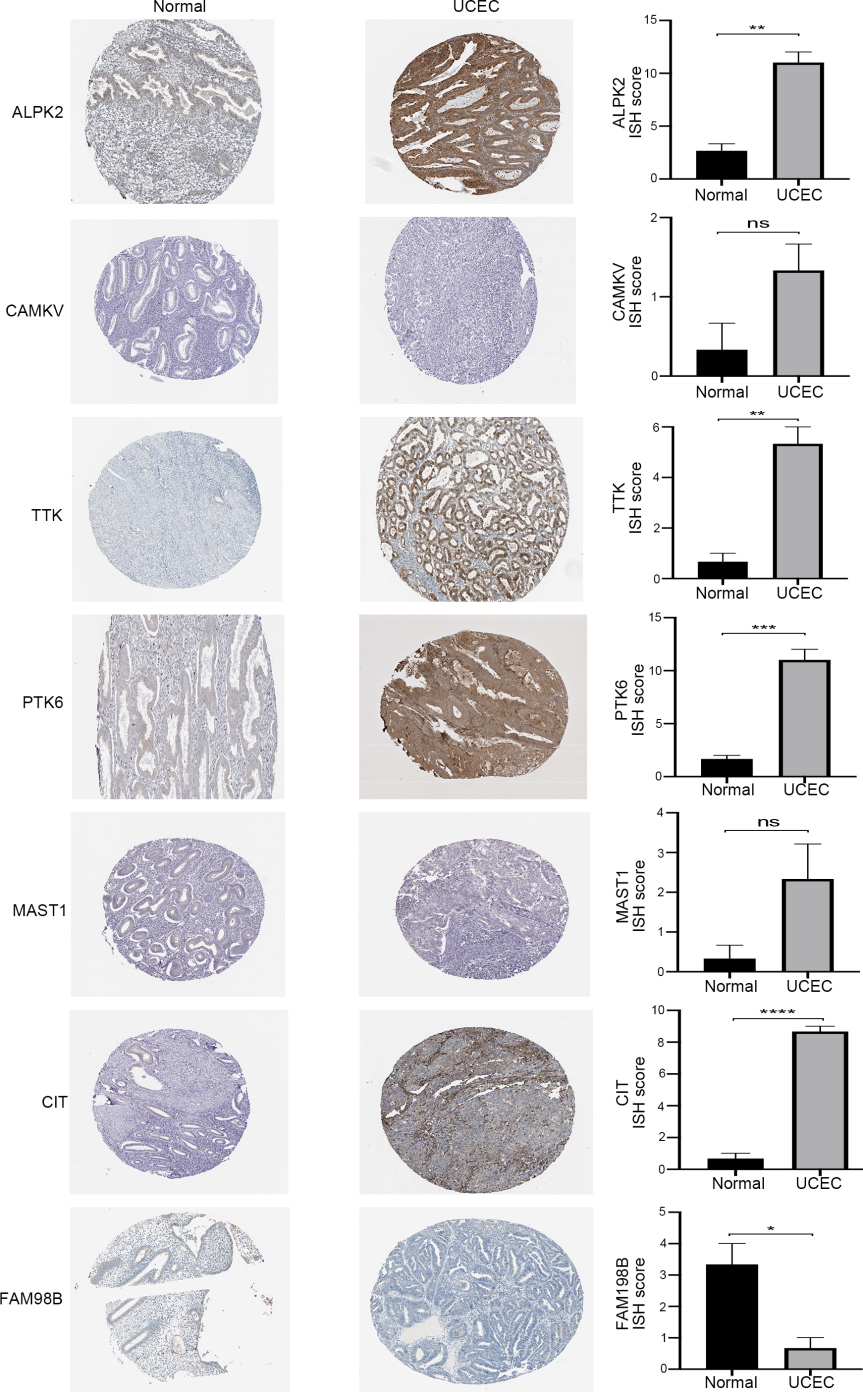
The immunohistochemical staining (left) and the corresponding histogram (right) of the seven prognostic kinase-related genes in normal endometrium and UCEC tissues. ns: not significant, *P<0.05,**P<0.01, ***P<0.001, ****P<0.0001.

The HPA contained the immunohistochemical results of 7 genes in UCEC tissues and normal endometrial tissues (**Figure 7**). Immunohistochemistry revealed ISH scores for four risk kinases (ALPK2, TTK, PTK6, and CIT) were significantly higher in endometrial carcinoma tissues than in healthy endometrium, which suggested that high expression of these genes may contribute to the progression of UCEC. Besides, same with qPCR results, the ISH score of FAM198B is lower in tumor specimens than in normal endometrium. These results suggested the potential feasibility of this signature for clinical usage.

### Analysis of tumor microenvironment and tumor immunity

**Figure 8:**
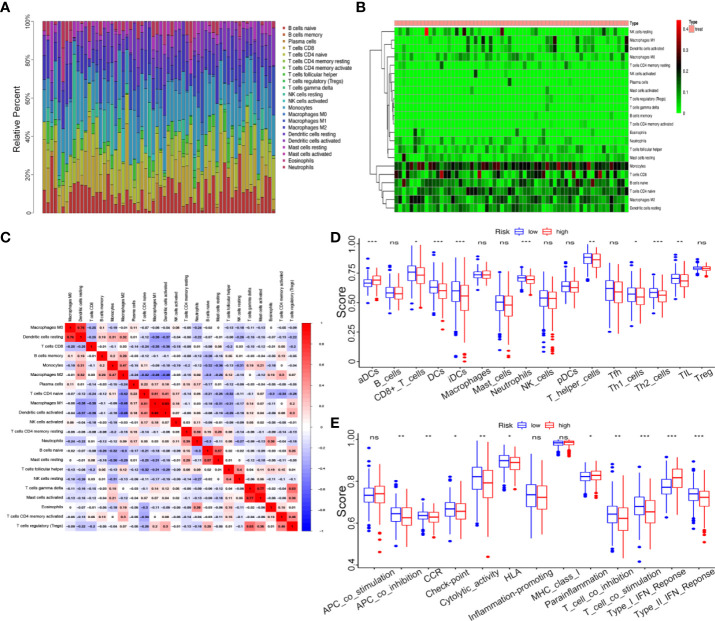
Correlation between kinome and TIC Infiltration. **(A)** Histogram of relative proportions of 22 tumor immune cells. **(B)** Heatmap of relative proportions of 22 tumor immune cells. **(C)** Correlation among 22 tumor immune cells (TICs). **(D)** The ssGSEA scores of 16 immune cells between the high- and low-risk groups. **(E)** The ssGSEA scores of 13 immune-related functions between different risk groups. ns, not significant, *P<0.05,**P<0.01, ***P<0.001.

To further explore the correlation between the kinome and TICs infiltration, we used the “CIBERSORT” algorithm to obtain and analyze the relative proportion of immune cells in the top 62 UCEC samples. The results were presented in the form of a bar plot and a heatmap (**Figures 8A, B**). As seen from the figures, the composition of TICs in the UCEC samples remained basically the same, mainly composed of monocytes, naive CD4 T cells, CD8 T cells, and naive B cells, with significant differences in some immune cells, such as M2 macrophages and dendritic cells. The associations between the 22 TIC proportions in 62 samples were represented using a correlation heatmap (**Figure 8C**).

To investigate the correlation between the risk score and immune status, we performed ssGSEA to quantify the relative abundance of diverse immune cell subpopulations, immune cell-related functions or pathways. Intriguingly, the enrichment of the TICs infiltration between the high- and low-risk groups exhibited significantly different dimensionalitiescontents, including the score of dendritic cells (DCs), aDCs, CD8^+^ T cells, iDCs, Neutrophils, T helper cells, Th1 cells, Th2 cells, and tumor-infiltrating lymphocytes (TIL) (P < 0.05, **Figure 8D**). For example, in low-risk groups, antitumor immune components such as CD8^+^ T cells, Th1 cells, and Th2 cells had a higher level, which meant lower-kinase risk patient showed stronger anti-tumor ability. Moreover, the scores of some immune functions, such as antigen-presenting cell (APC) co-inhibition, chemokine receptor (CCR), check-point, cytolytic activity, human leukocyte antigen (HLA), T cell co-inhibition, T cell co-stimulation, and Type II IFN response were significantly enriched in the low-risk group (**Figure 8E**).

## Discussion

Endometrial cancer is the sixth most common malignant tumor in women worldwide, and its incidence is still increasing in recent years (20). It is speculated that one-third of women will suffer from endometrial cancer during their lifetime (21, 22), thus threatening women’s health and life and increasing the social medical burden. Although the prognosis of endometrial cancer is not bad, the survival rate of advanced and special types of endometrial cancer is still poor (23), which may be attributed to a lack of reliable prognostic biomarkers. Recent studies have established a correlation between molecular markers such as autophagy genes (24), ferroptosis-related genes (25), immune-related long noncoding RNAs (lncRNAs) (26), and N6-methyladenosine-related lncRNAs (27), and UCEC prognosis, which may aid in determining clinical outcomes.

In the present study, we systematically investigated the expression of 538 kinome genes in UCEC tumor tissues and their associations with OS. We firstly constructed a novel prognostic model consisting of 7 kinase-related genes using univariate Cox and LASSO regression analyses. Among which, ALPK2, CAMKV, TTK, PTK6, MAST1, and CIT were highly expressed, meaning risk genes, whereas FAM198B was lowly expressed in the high-risk patients. By performing survival analysis on the training and testing sets and drawing ROC curves, we proved that our model can predict the prognosis for endometrial cancer patients based on risk factors and that the accuracy of the model was relatively high. Furthermore, according to the CIBERSORT algorithm and ssGSEA analysis, the association between the relative proportion of immune cells and kinome represented significant differences, including immune cell subpopulations and immune cell-related functions in the high- and low-risk groups.

ALPK2 (Alpha Kinase 2) is a member of an atypical alpha protein kinase family and could regulate cell cycle and DNA repair genes to participate in cancer development (28). It has known that the upregulation of ALPK2 is related to the progression of bladder cancer and renal cancer (29, 30). CAMKV (CaM Kinase Like Vesicle Associated), a pseudokinase of the CaMK family with unknown function, as a synaptic protein crucial for dendritic spine maintenance (31), is regulated by AMPA receptors (32). It is reported that CAMKV could as an immunotherapeutic target for MYCN amplified neuroblastoma (33). TTK (TTK Protein Kinase) enables to phosphorylate of tyrosine, serine, and threonine (34) and is associated with cell proliferation, precise division, DNA damage response, and organ development (35). In gastric cancer, TTK activates the Akt-mTOR pathway to regulate cell proliferation and apoptosis (36). PTK6 (Protein Tyrosine Kinase 6) is part of the PTK6 family of intracellular tyrosine kinases. Its expression is regulated by hypoxia and cell stress, and its kinase activity is induced by several growth factor receptors implicated in cancer including members of the ERBB family, IGFR1, and MET (37). PTK6 has been most well studied in breast cancer (38), prostate cancer (39), and multiple cancer. In most situations, increased PTK6 mRNA levels contribute to cancer cell migration, invasion, and metastases, and are associated with decreased survival, same with our study result. MAST1 (Microtubule Associated Serine/Threonine Kinase 1) belongs to a family containing four members, MAST1-MAST4. It is demonstrated that MAST1 rewires cRaf-Independent MEK activation to drive Cisplatin Resistance in Human Cancers (40). CIT (Citron Rho-Interacting Serine/Threonine Kinase) functions in cell division (41). Recent studies indicate that CIT plays a key role in the development of human cancer, including colon cancer (42) and bladder cancer (43). FAM198B (Family With Sequence Similarity 198 Member B), also named GASK1B (Golgi Associated Kinase 1B), is located in the Golgi apparatus. Based on the limited studies, FAM198B mainly acts as a tumor suppressor gene to take effects, for example, FAM198B blocks ERK-mediated MMP-1 expression to prolong survival and inhibit metastasis in lung adenocarcinoma (44), and our study provides evidence for this discovery.

Several studies have suggested a close correlation between kinase and tumor immune, such as tumor suppressor kinase DAPK3 regulating STING-IFN-β pathway to drive tumor-intrinsic immunity (45). In our study, when dividing the UCEC samples into low- and high-risk groups according to the kinase-related gene signature, we found that there were significant differences in the types of tumor-infiltrating immune cells and immune-related functions between them. The results revealed that the low-risk group had high levels of multiple antitumor immune components, including CD8^+^ T cells, Th1 cells, and Th2 cells, and scored higher on anti-tumor-related pathways, such as HLA (46) and T cell co-stimulation. Therefore, attenuated antitumor immunity in patients with high risk may be an explanation for their poor prognosis.

There are several limitations of this study. First, our prognostic model was based on public databases and is a retrospective study, which means it’s necessary to need more prospective real-world data to verify and improve its clinical utility. Second, we mainly focused on the kinase-related genes, the study Dimension is too narrow, so the intrinsic weakness of merely considering a single hallmark to build a prognostic model is inevitable. In addition, further studies are needed to assess the underlying mechanisms associated with inflammatory infiltration of the identified kinases in UCEC.

## Conclusion

In conclusion, based on seven selected kinase-related genes (ALPK2, CAMKV, TTK, PTK6, MAST1, CIT, and FAM198B), we identified and validated a prognostic signature possessing the independent predictive ability of UCEC diagnosis and immunotherapy status in TCGA datasets. This study offered a novel insight into the reliable integrated model according to the human kinome in UCEC prognostic prediction.

## Data availability statement

The datasets presented in this study can be found in online repositories. The names of the repository/repositories and accession number(s) can be found in the article/**Supplementary Material**.

## Ethics statement

The studies involving human participants were reviewed and approved by the Ethics Committee of Tongji Medical College, Huazhong University of Science and Technology. The patients/participants provided their written informed consent to participate in this study.

## Author contributions

SW conceived and designed the study. HW supervised the study. JZ and XC collected and analyzed the data. RS edited and wrote the manuscript. ZY revised and proofread the manuscript. All authors contributed to the article and approved the submitted version.

## Funding

This work is supported by the Major Technical Innovation Project in Hubei Province of China (Grant No. 2019ACA138).

## Acknowledgments

All authors appreciate the Cancer Genome Atlas and The Human Protein Atlas for the open data.

## Conflict of interest

The authors declare that the research was conducted in the absence of any commercial or financial relationships that could be construed as a potential conflict of interest.

## Publisher’s note

All claims expressed in this article are solely those of the authors and do not necessarily represent those of their affiliated organizations, or those of the publisher, the editors and the reviewers. Any product that may be evaluated in this article, or claim that may be made by its manufacturer, is not guaranteed or endorsed by the publisher.

## Supplementary material

Supplementary Table 1 Human kinome and the kinome plasmid library.

Supplementary Table 2 The sequence of qRT-PCR primers.

Supplementary Table 3 List of differentially expressed kinases between UCEC tissue and normal endometrial tissue in the TCGA dataset.

Supplementary Table 4 Gene ontology enrichment analysis of the differentially expressed kinases in the TCGA dataset.

Supplementary Table 5 KEGG enrichment analysis of the differentially expressed kinases in the TCGA dataset.

Supplementary Figure 1 Survival curves for 7 prognostic kinase-related genes.

## Abbreviations

UCEC, Uterine Corpus Endometrial Carcinoma; TCGA, The Cancer Genome Atlas; GO, Gene Ontology; KEGG, Kyoto Encyclopedia of Genes and Genomes; LASSO, Least Absolute Shrinkage and Selection Operator; ROC, Receiver operating characteristic; GAPDH, Glyceraldehyde 3-phosphate dehydrogenase; qRT-PCR, Quantitative real-time polymerase chain reaction; ISH, Immunohistochemistry; HPA, Human Protein Atlas; PPI, Protein-protein interaction; ALPK2, Alpha Kinase 2; CAMKV, CaM Kinase Like Vesicle Associated; TTK, TTK Protein Kinase; PTK6, Protein Tyrosine Kinase 6; MAST1, Microtubule Associated Serine/Threonine Kinase 1; CIT, Citron Rho-Interacting Serine/Threonine Kinase; FAM198B, Family With Sequence Similarity 198 Member B; OS, Overall survival; AUC, Area under the curve; TIC, Tumor-infiltrating immune cell; POLE, polymerase epsilon; MSI, microsatellite-instable; CN, copy number.
