# Supplementary figures and images for: Identification of an integrated kinase-related prognostic gene signature associated with tumor immune microenvironment in human uterine corpus endometrial carcinoma
Source: Front Oncol. 2022 Sep 7;12:944000. doi: 10.3389/fonc.2022.944000 (PMC9491090; doi:10.3389/fonc.2022.944000)

Supplementary Figure S1.

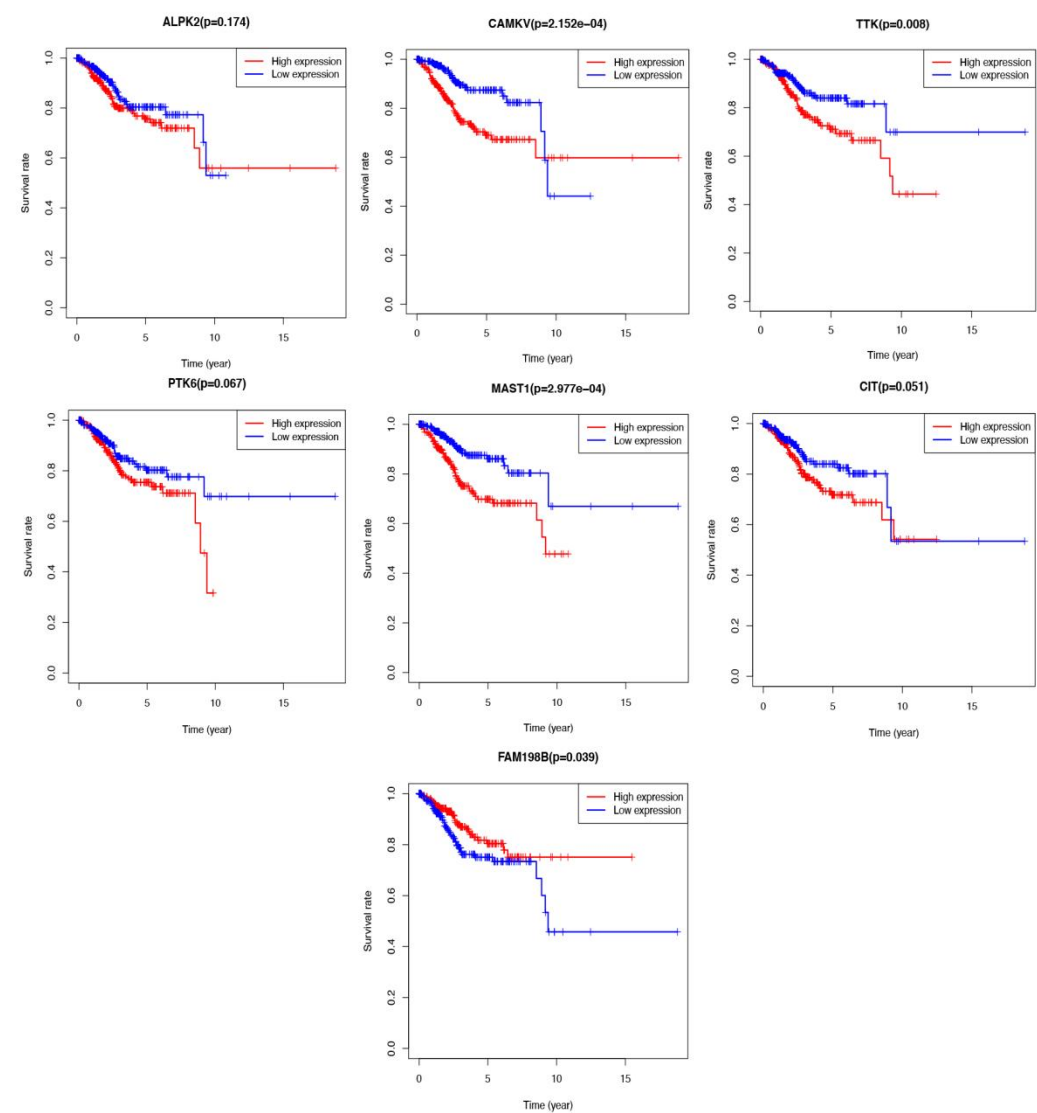

Survival curves for 7 prognostic kinase-related genes.

Supplement: Supplementary Figure 1 — Survival curves for 7 prognostic kinase-related genes. [file Image_1.pdf]
